# QSAR-Based Virtual Screening: Advances and Applications in Drug Discovery

**DOI:** 10.3389/fphar.2018.01275

**Published:** 2018-11-13

**Authors:** Bruno J. Neves, Rodolpho C. Braga, Cleber C. Melo-Filho, José Teófilo Moreira-Filho, Eugene N. Muratov, Carolina Horta Andrade

**Affiliations:** ^1^LabMol – Laboratory for Molecular Modeling and Drug Design, Faculdade de Farmácia, Universidade Federal de Goiás, Goiânia, Brazil; ^2^Laboratory of Cheminformatics, Centro Universitário de Anápolis (UniEVANGÉLICA), Anápolis, Brazil; ^3^Laboratory for Molecular Modeling, Division of Chemical Biology and Medicinal Chemistry, Eshelman School of Pharmacy, University of North Carolina at Chapel Hill, Chapel Hill, NC, United States; ^4^Department of Chemical Technology, Odessa National Polytechnic University, Odessa, Ukraine

**Keywords:** cheminformatics, machine learning, molecular descriptors, computer-assisted drug design, virtual screening

## Abstract

Virtual screening (VS) has emerged in drug discovery as a powerful computational approach to screen large libraries of small molecules for new hits with desired properties that can then be tested experimentally. Similar to other computational approaches, VS intention is not to replace *in vitro* or *in vivo* assays, but to speed up the discovery process, to reduce the number of candidates to be tested experimentally, and to rationalize their choice. Moreover, VS has become very popular in pharmaceutical companies and academic organizations due to its time-, cost-, resources-, and labor-saving. Among the VS approaches, quantitative structure–activity relationship (QSAR) analysis is the most powerful method due to its high and fast throughput and good hit rate. As the first preliminary step of a QSAR model development, relevant chemogenomics data are collected from databases and the literature. Then, chemical descriptors are calculated on different levels of representation of molecular structure, ranging from 1D to *n*D, and then correlated with the biological property using machine learning techniques. Once developed and validated, QSAR models are applied to predict the biological property of novel compounds. Although the experimental testing of computational hits is not an inherent part of QSAR methodology, it is highly desired and should be performed as an ultimate validation of developed models. In this mini-review, we summarize and critically analyze the recent trends of QSAR-based VS in drug discovery and demonstrate successful applications in identifying perspective compounds with desired properties. Moreover, we provide some recommendations about the best practices for QSAR-based VS along with the future perspectives of this approach.

## Introduction

Quantitative structure–activity relationship (QSAR) analysis is a ligand-based drug design method developed more than 50 years ago by Hansch and Fujita (1964). Since then and until now, QSAR remains an efficient method for building mathematical models, which attempts to find a statistically significant correlation between the chemical structure and continuous (pIC_50_, pEC_50_, Ki, etc.) or categorical/binary (active, inactive, toxic, nontoxic, etc.) biological/toxicological property using regression and classification techniques, respectively (Cherkasov et al., 2014). In the last decades, QSAR has undergone several transformations, ranging from the dimensionality of the molecular descriptors (from 1D to *n*D) and different methods for finding a correlation between the chemical structures and the biological property. Initially, QSAR modeling was limited to small series of congeneric compounds and simple regression methods. Nowadays, QSAR modeling has grown, diversified, and evolved to the modeling and virtual screening (VS) of very large data sets comprising thousands of diverse chemical structures and using a wide variety of machine learning techniques (Cherkasov et al., 2014; Mitchell, 2014; Ekins et al., 2015; Goh et al., 2017).

This review is devoted to (i) critical analysis of advantages and disadvantages of QSAR-based VS in drug discovery; (ii) demonstration of several successful QSAR-based discoveries of compounds with desired properties; (iii) description of best practices for the QSAR-based VS; and (iv) discussion of future perspectives of this approach.

## Best Practices in QSAR Modeling and Validation

High-throughput screening (HTS) technologies resulted in the explosion of amount of data suitable for QSAR modeling. As a result, data quality problem became one of the fundamental questions in cheminformatics. As obvious as it seems, various errors in both chemical structure and experimental results are considered as major obstacle to building predictive models (Young et al., 2008; Southan et al., 2009; Williams and Ekins, 2011).

Considering these limitations, Fourches et al. (2010; 2015; 2016) developed the guidelines for chemical and biological data curation as a first and mandatory step of the predictive QSAR modeling. Organized into a solid functional process, these guidelines allow the identification, correction, or, if needed, removal of structural and biological errors in large data sets. Data curation procedures include the removal of organometallics, counterions, mixtures, and inorganics, as well as the normalization of specific chemotypes, structural cleaning (e.g., detection of valence violations), standardization of tautomeric forms, and ring aromatization. Additional curation elements include averaging, aggregating, or removal of duplicates to produce a single bioactivity result. Detailed discussion of aforementioned data curation procedures can be found elsewhere (Fourches et al., 2010, 2015, 2016).

The Organization for Economic Cooperation and Development (OECD) developed a set of guidelines that the researchers should follow to achieve the regulatory acceptance of QSAR models. According to these principles, QSAR models should be associated with (i) defined end point, (ii) unambiguous algorithm, (iii) defined domain of applicability, (iv) appropriate measures of goodness-of-fit, robustness, and predictivity, and (v) if possible, mechanistic interpretation (OECD, 2004). In our opinion, the additional rule requesting thorough data curation as a mandatory preliminary step to model development should be added there.

## Continuing Importance of QSAR as Virtual Screening Tool

The current pipeline to discover hit compounds in early stages of drug discovery is a data-driven process, which relies on bioactivity data obtained from HTS campaigns (Nantasenamat and Prachayasittikul, 2015). Since the cost of obtaining new hit compounds in HTS platforms is rather high, QSAR modeling has been playing a pivotal role in prioritizing compounds for synthesis and/or biological evaluation. The QSAR models can be used for both hits identification and hit-to-lead optimization. In the latter, a favorable balance between potency, selectivity, and pharmacokinetic and toxicological parameters, which is required to develop a new, safe, and effective drug, could be achieved through several optimization cycles. As no compound need to be synthesized or tested before computational evaluation, QSAR represents a labor-, time-, and cost-effective method to obtain compounds with desired biological properties. Consequently, QSAR is widely practiced in industries, universities, and research centers around the world (Cherkasov et al., 2014).

**FIGURE 1:**
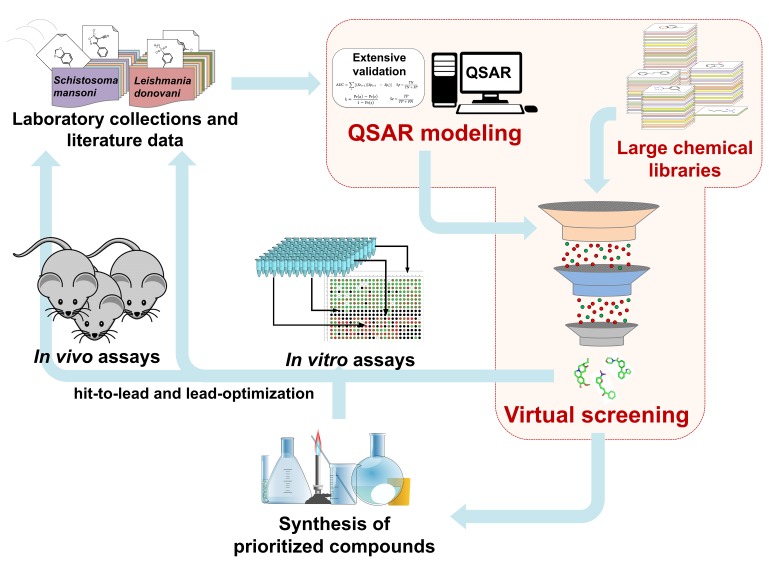
QSAR-based virtual screening workflow.

The general scheme of QSAR-based VS approach is shown in Figure 1. Initially, the data sets collected from external sources are curated and integrated to remove or correct inconsistent data. Using these data, QSAR models are developed and validated following OECD guidelines and best practices of modeling. Then, QSAR models are used to identify chemical compounds predicted to be active against selected endpoints from large chemical libraries (Cherkasov et al., 2014). In principle, VS is often compared to a funnel, where a large chemical library (i.e., 10^5^ to 10^7^ chemical structures) is reduced by QSAR models to a smaller number of compounds, which then will be tested experimentally (i.e., 10^1^ to 10^3^ chemical structures) (Kar and Roy, 2013; Tanrikulu et al., 2013). However, it is important to mention that modern VS workflows incorporate additional filtering steps, including: (i) sets of empirical rules [e.g., Lipinski’s (Lipinski et al., 1997) rules], (ii) chemical similarity cutoffs, (iii) other QSAR-based filters (e.g., toxicological and pharmacokinetic endpoints), and (iv) chemical feasibility and/or purchasability (Cherkasov et al., 2014). Although the experimental validation of computational hits does not represent part of the QSAR methodology, this should be performed as the final important step. After experimental validation, a multi-parameter optimization (MPO) with QSAR predictions of potency, selectivity, and pharmacokinetic parameters can be conducted. This information will be crucial during hit-to lead and lead optimization design of the compound series, to find the properties balance (potency, selectivity, and PK) related with the effect of different decoration patterns to establish a new series of target compounds for *in vivo* evaluation.

## QSAR-Based Virtual Screening vs. High-Throughput Screening

High-throughput screening can rapidly identify large subsets of molecules with desired activity from large screening collections of compounds (10^5^–10^6^ compounds) using automated plate-based experimental assays (Mueller et al., 2012). However, the hit rate of HTS ranges between 0.01% and 0.1% and this highlights the frequently encountered limitation that most of the screened compounds are routinely reported as inactive toward the desired bioactivity (Thorne et al., 2010). Consequently, the drug discovery cost increases according to the number of tested compounds (Butkiewicz et al., 2013). On the other hand, typical hit rates from a validated VS method, including QSAR-based, typically range between 1% and 40%. Thus, VS campaigns are found to have a higher rate of biologically active compounds and at a lower cost than HTS.

In this perspective, we show that QSAR-based VS could be used to enrich hit rates of HTS campaigns. For example, Mueller et al. (2010) employed both HTS and QSAR models to search novel positive allosteric modulators for mGlu_5_, a G-protein coupled receptor involved in disorders like schizophrenia and Parkinson’s disease. First, the HTS of approximately 144,000 compounds resulted in a total of 1,356 hits, with a hit rate of 0.94%. Then, this dataset was used to build continuous QSAR models (combining physicochemical descriptors and neural networks), which were subsequently applied to screen a database of approximately 450,000 compounds. Finally, 824 compounds were acquired for biological testing and 232 were confirmed as active (hit rate of 28.2%) (Mueller et al., 2010). In another study, Rodriguez et al. (2010) screened approximately 160,000 compounds to identify 624 antagonists of mGlu_5_. Further, these data were used to develop QSAR models and, then, applied to screen near 700,000 compounds from ChemDiv database. Among them, 88 of acquired compounds were active, corresponding to a hit rate of 3.6% while the HTS had a hit rate of 0.2% (Mueller et al., 2012).

## Practical Applications of QSAR-Based Virtual Screening

Despite its obvious advantages, QSAR modeling remains underestimated as a VS tool. Unfortunately, QSAR is still seen as a complementary analysis to studies of synthesis and biological evaluation, often introduced in the study without any justification or additional perspective. Despite the small number of VS applications available in the literature, most of them led to the discovery of promising hits and lead candidates. Below, we discuss some successful applications of QSAR-based VS for the discovery of new hits and hit-to-lead optimization.

### Malaria

Malaria is an infectious disease caused by five different species of *Plasmodium* parasites and transmitted to humans through the bite of infected female mosquitoes of the genus *Anopheles*. The most lethal species is *P. falciparum*, which can lead to severe illness and death (Phillips et al., 2017). Malaria is a widespread disease; 91 countries and areas have ongoing transmission. According to World Health Organization (WHO), about 216 million cases and 445,000 deaths from malaria were reported in 2016 (WHO, 2018c). Furthermore, the resistance to antimalarial drugs is a common and growing issue and constitutes a substantial threat for populations in endemic regions (Gorobets et al., 2017; Menard and Dondorp, 2017). In a study reported by Zhang et al. (2013), a data set of 3,133 compounds reported as active or inactive against *P. falciparum* chloroquine susceptible strain (3D7) was used to develop QSAR models. The models were built using Dragon descriptors (0D, 1D, and 2D), ISIDA-2D fragments descriptors and support vector machines (SVM) method. During QSAR modeling and validation, the data set was randomly divided into modeling and external evaluation set. Additionally, the modeling set was divided multiple times in training and test sets using the Sphere Exclusion algorithm. Then, by using a consensus approach, the QSAR models were applied for VS of the ChemBridge database. After VS, 176 potential antimalarial compounds were identified and submitted to experimental validation along with 42 putative inactive compounds, used as negative controls. Twenty-five compounds presented antimalarial activity in *P. falciparum* growth inhibition assays and low cytotoxicity in mammalian cells. All 42 compounds predicted as inactives by the models were confirmed experimentally (Zhang et al., 2013). The confirmed experimental hits presented new chemical scaffolds against *P. falciparum* and could be promising starting points for the development of new optimized antimalarial agents.

### Schistosomiasis

Schistosomiasis is a disease caused by flatworms of the genus *Schistosoma* that affects 206 million of people worldwide (WHO, 2018d). The current reliance on only one drug, praziquantel, for treatment and control of this disease calls for the urgent discovery of novel anti-schistosomal drugs (Colley et al., 2014). Aiming at discovering new drugs, our group developed binary QSAR models for *Schistosoma mansoni* thioredoxin glutathione reductase (*Sm*TGR), a validated target for schistosomiasis (Kuntz et al., 2007), to find new structurally dissimilar compounds with antischistosomal activity (Neves et al., 2016). To achieve this goal, we designed a study with the following steps: (i) curation of the largest possible data set of *Sm*TGR inhibitors, (ii) development of rigorously validated and mechanistically interpretable models, and (iii) application of generated models for VS of ChemBridge library. Using the QSAR models, we prioritized 29 compounds for further experimental evaluation. As a result, we found that the QSAR models were efficient for discovery of six novel hit compounds active against schistosomula and three hits active against adult worms (hit rate of 20.6%). Among them, 2-[2-(3-methyl-4-nitro-5-isoxazolyl)vinyl]pyridine and 2-(benzylsulfonyl)-1,3-benzothiazole, two compounds representing new chemical scaffolds have activity against schistosomula and adult worms at low micromolar concentrations and therefore represent promising antischistosomal hits for further hit-to-lead optimization (Neves et al., 2016).

In another study, we developed continuous QSAR models for a data set of oxadiazoles inhibitors of *sm*TGR (Melo-Filho et al., 2016). Using a combi-QSAR approach, we built a consensus model combining the predictions of individual 2D- and 3D-QSAR models. Then, the model was used for VS of ChemBridge database and the 10 top ranked compounds were further evaluated *in vitro* against schistosomula and adult worms. Additionally, we applied five highly predictive in-house QSAR models for prediction of important pharmacokinetics and toxicity properties of the new hits. The experimental results showed that 4-nitro-3,5-bis(1-nitro-1H-pyrazol-4-yl)-1H-pyrazole (LabMol-17) and 3-nitro-4-{[(4-nitro-1,2,5-oxadiazol-3-yl)oxy]methyl}-1,2,5-oxadiazole (LabMol-19), two compounds containing new chemical scaffolds (hit rate of 20.6%), were highly active in both life stages of the parasite at low micromolar concentrations (Melo-Filho et al., 2016).

### Tuberculosis

*Mycobacterium tuberculosis*, the causative agent of tuberculosis (TB), kills about 1.6 million people every year (WHO, 2018e). The current treatment of this disease takes approximately 9 months, which normally leads to noncompliance and, hence, the emergence of multidrug-resistant bacteria (AlMatar et al., 2017). Aiming the design of new anti-TB agents, our group used QSAR models to design new series of chalcone (1,3-diaryl-2-propen-1-ones) derivatives. Initially, we retrieved from the literature all chalcone compounds with *in vitro* inhibition data against *M. tuberculosis* H37Rv strain. After rigorous data curation, these chalcones were subject to structure–activity relationships (SAR) analysis. Based on SAR rules, bioisosteric replacements were employed to design new chalcone derivatives with optimized anti-TB activity. In parallel, binary QSAR models were generated using several machine learning methods and molecular fingerprints. The fivefold external cross-validation procedure confirmed the high predictive power of the developed models. Using these models, we prioritized series of chalcone derivatives for synthesis and biological evaluation (Gomes et al., 2017). As a result, five 5-nitro-substituted heteroaryl chalcones were found to exhibit MICs at nanomolar concentrations against replicating mycobacteria, as well as low micromolar activity against nonreplicating bacteria. In addition, four of these compounds were more potent than standard drug isoniazid. The series also showed low cytotoxicity against commensal bacteria and mammalian cells. These results suggest that designed heteroaryl chalcones, identified with the help of QSAR models, are promising anti-TB lead candidates (Gomes et al., 2017).

### Viral Infections

Yearly, influenza epidemics can seriously affect all populations in the world. These annual epidemics are estimated to result in about 5 million cases and 650,000 deaths (WHO, 2018b). Influenza virus is mutating constantly, resulting in novel resistant strains, and hence, the development of new anti-influenza drugs active against these new strains is important to prevent pandemics (Laborda et al., 2016). Aiming the discovery of new anti-influenza A drugs, Lian et al. (2015) built binary QSAR models, using SVM and Naïve Bayesian methods, to predict neuraminidase inhibition, a validated protein target for influenza. Then, four different combinations of machine learning methods and molecular descriptors were applied to screen 15,600 compounds from an in-house database, among which 60 compounds were selected to experimental evaluation on neuraminidase activity. Nine inhibitors were identified, five of which were oseltamivir derivatives exhibiting potent neuraminidase inhibition at nanomolar concentrations. Other four active compounds belonged to novel scaffolds, with potent inhibition at low micromolar concentrations (Lian et al., 2015).

According to WHO, approximately 35 million people are infected with HIV (WHO, 2018a). The treatment for HIV infections requires a lifelong antiretroviral therapy, targeting different stages of HIV replication cycle. Consequently, because of the emergence of resistance and the lack of tolerability, development of novel anti-HIV drugs is of high demand (Cihlar and Fordyce, 2016; Garbelli et al., 2017). With the purpose of discovering new anti-HIV-1 drugs, Kurczyk et al. (2015) developed a two-step VS approach to prioritize compounds against HIV integrase, an important target to viral replication cycle. The first step was based on binary QSAR models, and the second on privileged fragments. Then, 1.5 million of commercially available compounds were screened, and 13 compounds were selected to be tested *in vitro* for inhibiting HIV-1 replication. Among them, two novel chemotypes with moderate anti-HIV-1 potencies were identified, and therefore, represent new starting points for prospective structural optimization studies.

### Mood and Anxiety Disorders

The 5-hydroxytryptamine 1A (5-HT_1A_) serotonin receptor has been an attractive target for treating mood and anxiety disorders such as schizophrenia (Nichols and Nichols, 2008; Lacivita et al., 2012). However, the currently marketed drugs targeting 5-HT_1A_ receptor possess severe side effects. To address this, Luo et al. (2014) developed a QSAR-based VS workflow to find new hit compounds targeting 5-HT_1A_ receptor. First, binary QSAR models were generated using Dragon descriptors and several machine learning methods. Then, developed QSAR models were rigorously validated and applied in consensus for VS four commercial chemical databases. Fifteen compounds were selected for experimental testing, and nine of them have proven to be active at low nanomolar concentrations. One of the confirmed hits, [(8α)-6-methyl-9,10-didehydroergolin-8-yl]methanol), showed very high binding affinity (Ki) of 2.3 nM against 5-HT_1A_ receptor.

### Future Directions and Conclusion

To summarize, we would like to emphasize that QSAR modeling represents a time-, labor-, and cost-effective tool to discover hit compounds and lead candidates in the early stages of drug discovery process. Analyzing the examples of QSAR-based VS available in the literature, one can see that many of them led to the identification of promising lead candidates. However, along with success stories, many QSAR projects fail on the model building stage. This is caused by the lack of understanding that QSAR is highly interdisciplinary and application field as well as general ignorance of the best practices in the field (Tropsha, 2010; Ban et al., 2017). Earlier, we have explained this by the undesirably high population of “button pushers,” that is, researchers who conduct modeling without understanding and analyzing the data and modeling process itself (Muratov et al., 2012). This was also explained by the elusive ease of obtaining computational model and making even advanced calculations without understanding of the sense and limitations of the approach (Bajorath, 2012). In addition to this, a lot of even experienced researchers target their efforts to a “vicious statistical cycle,” which main goal is to validate models using as many metrics as possible. In this case, the QSAR modeling is restricted to a single simple question: “What is the best metrics or the best statistical method”? Although we recognize that the right choice of statistical approach and especially rigorous external validation are necessary and represent an essential step in any computer-aided drug discovery study, we want to reinforce that QSAR modeling is useful only if it is applied for the solution of a formulated problem and results in development of new compounds with desired properties.

As future directions, we would like to point out that the era of big data has just started, and it is still in the chemical/biological data accumulation stage. Therefore, to avoid the situation that the number of assayed compounds available on literature exceeds the modeling capability, the development, and implementation of new machine learning algorithms and data curation methods capable of handling millions of compounds are urgently needed. Finally, the overall success of any QSAR-based VS project depends on the ability of a scientist to think critically and prioritize the most promising hits according to his experience. Moreover, the success rate of collaborative drug discovery projects, where the final selection of computational hits is done by both a modeler and an expert in a given field, is much higher than success rate of the projects driven solely by computational or experimental scientists.

## Author Contributions

All authors listed have made a substantial, direct and intellectual contribution to the work, and approved it for publication.

## Conflict of Interest Statement

The authors declare that the research was conducted in the absence of any commercial or financial relationships that could be construed as a potential conflict of interest.
